# 利妥昔单抗为基础治疗儿童再生障碍性贫血单倍体移植后EB病毒相关淋巴细胞增殖性疾病的前瞻性单中心临床研究

**DOI:** 10.3760/cma.j.cn121090-20231229-00345

**Published:** 2024-07

**Authors:** 枫 张, 冠华 胡, 盼 锁, 郑丽 徐, 露 柏, 慧芳 王, 雅美 黄山, 兰平 许, 英军 常, 晓辉 张, 晓军 黄, 翼飞 程

**Affiliations:** 北京大学人民医院，北京大学血液病研究所，国家血液系统疾病临床医学研究中心，造血干细胞移植北京市重点实验室，北京 100044

## Abstract

EB病毒（EBV）相关移植后淋巴细胞增殖性疾病（PTLD）是造血干细胞移植后最严重并发症之一。本研究纳入31例再生障碍性贫血（AA）行单倍体移植后发生PTLD的病例，归纳其临床特点，并按照利妥昔单抗治疗1次后是否能下降1个log分为利妥昔单抗单药治疗组或联合治疗组。AA患儿移植后PTLD发生率为10.16％，年龄>10岁者PTLD发生率明显升高（*χ*^2^＝11.336，*P*＝0.010）。31例患者中临床诊断27例，病理确诊4例。利妥昔单抗单药治疗组15例，联合治疗组15例。最终27例患儿治愈。3例患儿死亡，2年总生存率为（89.7±5.6）％。标准预处理方案、EBV复阳是影响PTLD预后的危险因素。两种治疗方案对预后影响差异无统计学意义。

## Abstract (en)

Epstein-Barr virus（EBV）associated post-transplant lymphoproliferative disorders（PTLD）are one of the most severe complications after hematopoietic stem cell transplantation（HSCT）. This study includes 31 cases of aplastic anemia（AA）patients who developed PTLD after haploidentical transplantation, summarizing their clinical characteristics and categorizing them into either rituximab monotherapy group or combination therapy group based on whether their condition improved by 1 log after a single dose of rituximab. The incidence of PTLD after HSCT in children with AA was 10.16％, and the incidence of PTLD in patients with age >10 years was significantly increased（*χ*^2^＝11.336, *P*＝0.010）. Of the 31 patients, 27 were clinically diagnosed and 4 were pathologically confirmed. Finally, 15 patients were classified into the rituximab treatment group and 15 patients into the combination treatment groups. Finally three patients died, and the 2-year overall survival rate was（89.7±5.6）％. Standard pre-treatment protocols and EBV reactivation are risk factors affecting the prognosis of PTLD. There was no statistically significant difference in the impact of the two treatment schemes on prognosis.

EB病毒（EBV）相关移植后淋巴细胞增殖性疾病（PTLD）是造血干细胞移植（HSCT）后的一种严重并发症。HSCT后免疫抑制，EBV特异性T细胞缺失或功能受损，EBV诱发淋巴细胞增殖从而导致PTLD。不同原发病、不同移植类型PTLD发生率不尽相同，介于1.2％～11.2％[1]。欧洲白血病感染会议（ECIL-6）及2022版中国专家共识均提出，PTLD的高危因素包括原发病为重型再生障碍性贫血（SAA）、T细胞去除（体内或体外）、EBV-IgG供受者不合、人类白细胞抗原（HLA）不合，移植物抗宿主病（GVHD）、巨细胞病毒（CMV）血症等[2]。

HSCT是根治儿童SAA最有效的办法，在无HLA全相合供者时，通常以单倍体移植作为替代选择，国内主流的预处理方案为抗胸腺细胞球蛋白（ATG）介导的体内去除T细胞，故此类患儿是PTLD发生的高危人群。然而既往儿童PTLD报道少，多局限于allo-HSCT后PTLD总体的发生及转归情况，且原发病混杂[3]–[5]。目前并无SAA患儿行单倍体移植后PTLD的单独报道。本研究参考我中心PTLD的治疗方案[6]，纳入自2016年1月1日起我中心所有SAA患儿行单倍体移植后诊断为PTLD的病例，依据危险度进行分层治疗，并评估治疗有效性、安全性，为此类患儿的诊疗提供参考依据。

## 病例与方法

一、病例资料

分析2016年1月1日至2023年8月1日北京大学人民医院血液科收治的所有行单倍体移植的310例SAA患儿（年龄≤18岁）的病例资料。最终31例确诊和临床诊断的PTLD患儿纳入本研究。本项研究通过北京大学人民医院伦理委员会审批（2023PHB204-001）。所有患儿的监护人均知情同意。

二、预处理方案及GVHD预防

无心脏毒性危险因素的患者接受改良白消安/环磷酰胺（Bu-Cy）联合兔源ATG清髓预处理方案。有心脏毒性危险因素的患者接受Bu-Cy-Flu联合ATG的减低剂量预处理方案[7]。GVHD预防方案为环孢素A（CsA）、霉酚酸酯（MMF）联合甲氨蝶呤（MTX）[8]。移植物：骨髓联合外周血干细胞；输注单个核细胞（mononuclear cell，MNC）中位数为10.18（7.05～18.72）×10^8^/kg，输注CD34^+^细胞中位数为3.46（1.13～10.94）×10^6^/kg。

三、EBV监测

移植后1个月内或中性粒细胞植入后每周监测EBV DNA拷贝数至移植后第100天。从第100天到第180天，每2周检测受体EBV-DNA拷贝数1次。外周血血浆EBV DNA载量至少1次大于1×10^3^拷贝数/ml诊断为EBV感染。

四、定义

PTLD的诊断遵循《造血干细胞移植后EB病毒相关淋巴增殖性疾病中国专家共识（2022年版）》[2]，临床诊断的PTLD定义为EBV阳性或组织样本中发现EBV感染，同时有发热、淋巴结肿大、肝脾肿大或其他终末器官受累表现。确诊的PTLD病理诊断应符合以下标准：①原有组织结构被增生的淋巴组织破坏；②采用细胞和（或）病毒标志物发现单克隆或寡克隆细胞群；③组织样本中发现EBV感染的证据；满足①或②任意1条及③为病理确诊。GVHD判定遵循参考文献[9]。粒细胞植活定义为连续3 d中性粒细胞计数>0.5×10^9^/L。血小板植活定义为连续7 d PLT>20×10^9^/L且脱离血小板输注。CMV阳性定义为血浆检测到CMV DNA>500拷贝数/ml。PTLD的CR定义为EBV-DNA阴性且无临床症状、体征，CT评估靶病灶完全消失。总生存（OS）时间定义为从移植至末次随访或任意原因的死亡。

五、PTLD治疗管理

参照我中心既往报道[6]，所有符合PTLD临床诊断者CsA均适度减量，外周血血浆EBV-DNA>1×10^3^拷贝数/ml即开始应用利妥昔单抗（375 mg/m^2^每周1次）治疗，直到EBV-DNA被清除，应用3～4剂。1剂利妥昔单抗后EBV-DNA下降1 log者纳入A组，监测EBV-DNA下降<1 log或疾病进展纳入B组并加用联合治疗方案，包括应用甲泼尼龙1～2 mg·kg^−1^·d^−1^、EBV特异性供者淋巴细胞回输、化疗等。利妥昔单抗回输后监测外周血CD19^+^ B细胞和免疫球蛋白IgG恢复情况。

六、统计学处理

采用SPSS 26.0进行统计分析。连续变量的比较采用Mann-Whitney *U*检验，分类资料的比较采用卡方检验或Fisher确切概率法，生存分析采用Kaplan-Meier法，多因素分析采用Cox回归模型。双侧*P*<0.05为差异有统计学意义。

## 结果

一、PTLD的发生情况

共310例AA患儿行单倍体移植，5例在回输干细胞期间出现心脏损伤并死亡，长期随访305例。其中男180例，女125例，HSCT时中位年龄9（1～18）岁。诊断PTLD者共31例。本中心自2022年8月起患儿粒细胞植入后开始应用来特莫韦预防CMV感染。单因素分析显示口服莱特莫韦者PTLD发生率是未口服者的2倍（20.0％对9.7％）。年龄>10岁者PTLD的发生率显著增高（*χ*^2^＝11.336，*P*＝0.01）。性别与PTLD的发生无关（*χ*^2^＝0.074，*P*＝0.786）。

二、PTLD患儿临床特点

共31例患儿诊断PTLD，其中男19例，女12例，中位发病年龄为11（2～18）岁，原发病确诊至HSCT的中位时间为25（1～171）个月，除1例植入不良外，所有患儿均获得植活，其中中位粒细胞植活时间为+12（+10～+21）d，中位血小板植活时间为+13（+8～+65）d。诊断PTLD的中位时间为+51（+18～+207）d。

所诊断患儿中4例为病理诊断，其余27例为临床诊断。临床表现上，22例（70％）以发热为主要表现，2例（7％）中枢神经系统（CNS）受累者表现为惊厥发作，2例（7％）肺部受累者以喘憋为主要症状，1例以“打鼾”为主要症状，1例扁桃体受累者以“咽痛”为主要症状，1例肠道受累者以腹痛、腹泻为主要表现。体格检查发现浅表淋巴结肿大者20例（64％），淋巴结合并其他器官受累者共5例（16％），分别为肝大、脾大或扁桃体肿大。单独终末器官受累者7例（22％），分别为CNS（1例）、肝脏（1例）、肠道（1例）、肺（3例）及扁桃体（1例）。合并CMV感染者16例（51％），所有患儿均检测到EBV阳性，其中血浆EBV DNA阳性者28例（90％），起病时中位EBV DNA为3.56（1.07～32.50）×10^3^拷贝数/ml，1例为肠道活检病理EBV原位杂交（EBER）阳性，1例为脑脊液EBV拷贝数阳性，1例为外周血二代测序检测到EBV。

三、PTLD患儿病理结果

共4例患者为病理确诊。例1为浆细胞型。骨髓免疫分型示CD38^st+^CD138^+^细胞占7.92％，表达CD19、CD38、CD138、c/mκ，不表达c/mλ、CD56、CD20、CD22、CD23，为异常克隆性浆细胞。骨髓活检免疫组化EBER阳性。例2行淋巴结活病理免疫组化示CD20、PAX-5部分+、CD3、CD5部分+、CD23（−）、CD10（−）、BCL-6（−）、CD38（+）、CD138（+）、Kappa、Lambda（+）、Ki-67（90％）、EBER阳性。例3淋巴结活检病理免疫组化示CD20部分+、PAX-5灶+、CD3散在+、CD5−、CD23−、Cyclin D1（−）、CD138散在+、MPO灶+、CD43部分+、CD34（−）、CD10（−）、Ki-67（70％）、EBER阳性。例4为淋巴结活检确诊：免疫组化示B细胞表型，具有不典型传染性单核细胞增多症样的部分特征和弥漫大B细胞淋巴瘤的部分特征，EBER阳性。

四、PTLD的治疗及危险因素分析

1例未予利妥昔单抗治疗，起病时EBV DNA为9.24×10^3^拷贝数/ml，EBV血症持续时间长，87 d后EBV转阴。余30例患儿均给予以利妥昔单抗为基础的治疗，起病时中位EBV拷贝数为3.055×10^3^拷贝数/ml，中位EBV转阴天数为8 d。利妥昔单抗1剂后EBV下降高于1个log纳入A组。A组患儿15例，病初中位EBV DNA为2.90×10^3^拷贝数/ml。利妥昔单抗1剂后EBV下降低于1个log或疾病进展纳入B组。B组患儿15例，中位EBV DNA为3.52×10^3^拷贝数/ml，两组起病时EBV DNA拷贝数差异无统计学意义（*P*＝0.771）。单因素分析显示利妥昔单抗治疗是否敏感与患儿年龄，性别，供者性别，血型是否相合，PTLD的发生时间，是否存在CMV感染和多部位感染，回输的MNC和CD34^+^细胞数，中性粒细胞、血小板植活时间以及是否存在Ⅱ度以上GVHD以及EBV转阴天数无关（*P*>0.05）。A组患儿体温恢复正常，应用利妥昔症状消失的中位剂量为1（1～2）剂，利妥昔单抗总剂量为3（1～4）剂，1例EBV复阳，予EBV-CTL回输治愈，所有患儿均无病生存。B组患儿在输注第2剂单抗时加用二线治疗，其中3例应用EBV-CTL回输，余11例加用甲泼尼龙1～2 mg/kg治疗，除2例治疗无效死亡，1例死于肺部感染外，应用利妥昔症状消失的中位剂量为2（1～4）剂，利妥昔单抗中位总剂量为3.5（2～4）剂。

五、长期随访和转归

中位随访24（1～85）个月，3例患者死亡，2例死于PTLD，1例死于闭塞性细支气管炎及呼吸心跳骤停。2年OS率为（89.7±5.6）％。单因素分析显示A组患儿3年OS率高于B组患儿［100.0％对（79.4±10.4）％］。起病年龄、性别、供者性别、血型是否相同、回输MNC和CD34^+^细胞数量、是否合并Ⅱ～Ⅳ度GVHD、是否应用莱特莫韦预防CMV、是否合并CMV感染、EBV转阴天数等均对OS无影响（*P*>0.1），而选择标准预处理方案、EBV复阳是OS的显著影响因素（*P*<0.05，详见表1）。由于本研究例数较少，将*P*<0.1的因素纳入多因素Cox回归，未发现影响PTLD预后的独立危险因素。

**表1:** 影响再生障碍性贫血单倍体移植后EB病毒相关PTLD患儿2年总生存（OS）的单因素分析

变量	例数	2年OS率（%）	统计量	*P*值
受者性别			1.719	0.190
男	19	100.0		
女	11	84.2±8.4		
年龄分组			2.627	0.105
≥10岁	22	95.5±4.4		
<10岁	8	72.9±16.5		
供者性别			0.811	0.368
男	24	87.1±7.0		
女	6	100.0		
血型			0.592	0.442
相合	16	93.8±6.1		
不合	14	84.4±10.2		
预处理方案			4.314	0.038
标准剂量方案	13	76.2±12.1		
减低剂量方案	17	100.0		
MNC计数			0.159	0.690
≥10.18×10^8^/kg	17	88.2±7.8		
<10.18×10^8^/kg	13	91.7±8.0		
CD34^+^细胞数			0.108	0.781
≥3.46×10^6^/kg	18	88.1±7.9		
<3.46×10^6^/kg	12	91.7±8.0		
Ⅲ~Ⅳ度GVHD			0.117	0.732
是	7	85.7±13.2		
否	23	91.3±5.9		
口服来特莫韦			1.044	0.307
是	8	100.0		
否	22	86.4±7.3		
合并CMV感染			2.665	0.103
是	16	81.3±9.8		
否	14	100.0		
治疗方案			3.235	0.072
利妥昔单抗单药治疗	15	100.0		
利妥昔单抗联合治疗	15	79.4±10.6		
EBV转阴时间			0.319	0.572
≥8 d	16	85.7±8.3		
<8 d	14	92.9±6.9		
起病EBV DNA			3.235	0.072
≥3.055×10^3^拷贝数/ml	15	79.4±10.6		
<3.055×10^3^拷贝数/ml	15	100.0		
诊断类型			0.756	0.385
临床诊断	25	91.6±5.7		
病理诊断	5	80.0±17.9		
EBV复阳			16.807	<0.001
是	3	33.3±27.2		
否	27	96.0±3.9		

**注** PTLD：移植后淋巴细胞增殖性疾病；MNC：单个核细胞；GVHD：移植物抗宿主病；CMV：巨细胞病毒；EBV：EB病毒

六、不良反应

所有患儿利妥昔单抗输注时均未观察到过敏反应。20例患儿应用利妥昔单抗1个月后外周血CD19^+^ B细胞/MNC为0（0～0.24％），低于正常范围。未发生利妥昔相关的影响长期生存的严重不良事件。

## 讨论

本研究展示了单倍体移植治疗儿童SAA后PTLD的发生率、临床特点、治疗方案、治疗效果和长期随访结果，是儿童AA后合并PTLD最大例数的报道。应用利妥昔单抗治疗成人EBV感染及PTLD国内已有专家共识，然而利妥昔单抗联合其他方案治PTLD，目前报道不多，还处于临床试验阶段[10]。儿童PTLD由于发病率低，目前报道少且国际上也没有统一的治疗方案[11]。本研究发现对于单独应用利妥昔单抗治疗不敏感的PTLD患儿，及时加用二线治疗后两组患儿的预后指标差异无统计学意义。本中心儿童AA行haplo-HSCT后PTLD的发生率为10.16％，高于国内闫洪敏等[3]报道，可能与闫洪敏等报道的患者部分为全相合移植有关。既往PTLD的报道显示PTLD发生的危险因素与年轻患者、应用ATG、非清髓预处理方案、免疫延迟、CMV感染有关[12]–[14]。本研究发现在儿童人群中，大年龄儿童（年龄>10岁）PTLD的发生率明显升高（*P*<0.001）。PTLD的临床表现多种多样，既往PTLD报道的受累部位集中于淋巴结、扁桃体等，临床多伴有发热，也有结外受累表现[15]，CNS受累也有病例报道[16]。本研究中8例（30％）PTLD患儿无发热表现，仅以肝脾大、淋巴结或结外受累为首发表现。

Zhu等[17]报道了26例包含应用北京方案预处理后PTLD的成人和儿童患者，1年OS率为46.8％，PTLD较低的OS率与起病时EBV高拷贝（>10^6^拷贝数/ml）、1剂或2剂利妥昔单抗后EBV仍阳性、是否合并多重感染相关。国内王春静等[5]报道的13例儿童PTLD的病例，OS率为84.6％，2例死亡病例均合并了多重感染。同王春静等研究相似，本研究OS率为88.9％。既往报道的全血EBV DNA>1×10^5^拷贝数/ml时应用利妥昔单抗可预防PTLD[17]。最近的研究提出血浆EBV DNA>1×10^3^拷贝数/ml或全血EBV DNA>5×10^3^拷贝数/ml即可应用利妥昔单抗抢先治疗预防PTLD[18]。本研究采用血浆EBV DNA拷贝数为监测指标，发现EBV血症进展为PTLD后启动利妥昔单抗仍可获得较好预后。尽管本中心PTLD患者长期生存率明显提高，但本研究发现PTLD病初EBV的高拷贝可能与不良预后相关（*P*＝0.072），提示EBV高拷贝（>3.055×10^3^拷贝数/ml）时需要更积极的干预措施。另外，本研究发现减低预处理患者PTLD的死亡率低于标准预处理患者，显示减低预处理方案在PTLD后的长期生存中起到了更积极的作用。而血浆EBV DNA经治疗后复阳也是影响预后的危险因素（*P*<0.001），预示EBV复阳人群预后更差。

综上，基于利妥昔单抗为基础的方案治疗HSCT后的PTLD，其长期生存高于既往报道，且未发现严重不良反应[19]。

本研究存在一定的局限性，因时间跨度长，早年病例并未进行感染EBV淋巴细胞分选，后续会加入EBV淋巴细胞分选结果并积累病例加以评估疗效，完善后续随访。
